# Positioning Locality Using Cognitive Directions Based on Indoor Landmark Reference System

**DOI:** 10.3390/s18041049

**Published:** 2018-03-31

**Authors:** Yankun Wang, Hong Fan, Ruizhi Chen, Huan Li, Luyao Wang, Kang Zhao, Wu Du

**Affiliations:** 1 State Key Lab for Information Engineering in Surveying, Mapping and Remote Sensing, Wuhan University, 129 Luoyu Road, Wuhan 430079, China; yankun.wang@whu.edu.cn (Y.W.); ruizhi.chen@whu.edu.cn (R.C.); wangluyao@whu.edu.cn (L.W.); kzhao@whu.edu.cn (K.Z.); d.y.wu.du@gmail.com (W.D.); 2 Collaborative Innovation Center of Geospatial Technology, Wuhan University, 129 Luoyu Road, Wuhan 430079, China; 3 State Key Laboratory of Hydroscience and Engineering, Tsinghua University, Beijing 100084, China; lihuan2016@tsinghua.edu.cn; 4 Department of Hydraulic Engineering, Tsinghua University, Beijing 100084, China

**Keywords:** locality description, positioning locality indoors, uncertainty, spatial relations, indoor landmark reference system

## Abstract

Locality descriptions are generally communicated using reference objects and spatial relations that reflect human spatial cognition. However, uncertainty is inevitable in locality descriptions. Positioning locality with locality description, with a mapping mechanism between the qualitative and quantitative data, is one of the important research issues in next-generation geographic information sciences. Spatial relations play an important role in the uncertainty of positioning locality. In indoor landmark reference systems, the nearest landmarks can be selected when describing localities by using direction relations indoors. By using probability operation, we combine a set of uncertainties, that is, near and direction relations to positioning locality. Some definitions are proposed from cognitive and computational perspectives. We evaluate the performance of our method through indoor cognitive experiments. Test results demonstrate that a positioning accuracy of 3.55 m can be achieved with the semantically derived direction relationships in indoor landmark reference systems.

## 1. Introduction

Geographic information sciences (GIS) have been entering an era of information explosion. The data-related geographic can be divided into many classes, according to their sources and format, such as raster dataset, shape file, textual information, and voice [1]. Locality description, which is a common form of voice, conveys considerable spatial information and can be derived from our daily communication. The issue of dealing with the locality description information is a research hot spot of next-generation GIS for many scholars [2,3,4,5].

Locality description reflects direct or indirect human interaction with environment directly [6]. As an external expression of cognition, the uncertainty that is associated with locality description is inevitable [7]. Locality description generally contains spatial relationships (i.e., topological, distance, and direction relations) and reference objects (ROs). Any feature with a name can be regarded as an RO [8,9]. Topological relations, which convey rough information-related locality and can be refined by distance or direction relations, are seldom used directly in locality description positioning [10]. The distance and direction relations are usually combined to describe locality, which conveys many clues to position locality [10].

Humans have a weak sense of direction indoors, and relative directions are used frequently in locality description. For example, locality description indoors can be given as follows: “Object A is in front of me, and object B is on my left”. The locality description is complex, either explicitly or implicitly [11], especially in a landmark reference system [12] (i.e., a reference system where people can describe his locality with one or several landmarks), in which the nearest landmark can be selected easily to describe locality [12]. On the basis of this concept, the locality description (“Object A is in front of me, and object B is on my left”) stated above in an indoor landmark reference system (ILRS) implies that objects A and B are near the individual. Hence, the meaning of “Object A is in front of me, and object B is on my left” in ILRS is the same as that of “Object A is in front of me, object B is on my left, and they are all near to me”. This paper introduces a novel method of positioning localities indoors by using locality description in ILRS. 

Many related works in the literature provide meaningful references. The conceptual function between the membership degree of “near” and the distance between objects is defined in [10], but no related practical application is discussed. In computational geometry, the near relation can be represented by Voronoi diagrams [13]. Gong [12,14] proposed a mixed-selection probability function that was based on Euclidean distance and Voronoi stolen area to model near relations. However, this function focused only on points and provided no further discussion about polygons. Nevertheless, this function provided considerable inspiration for related studies. The human perception of direction, whether absolute or relative, is closely related to angular information. As stated in [15], the membership functions about “left of”, “right of”, “above”, and so on, are defined. They all relate to angular information but differ in parameters. The function has been developed in accordance with different applications [15,16]. 

The contributions of this work are as follows:

(1) On the basis of the complexity of locality description, we propose that people tend to select near landmarks in ILRS when describing locality with the directions of locality description.
(2) We develop a novel membership function for polygon landmarks to model qualitative distance relations, such as near relations.
(3) We propose the calculation of relative direction for polygon landmarks from the perspectives of algorithm and cognition.
(4) We provide the method of positioning locality based on a joint probability function that consists of qualitative distance and relative direction membership functions. Cognitive experiments are conducted to evaluate the positioning accuracy. Test results demonstrate that a positioning accuracy of 3.55 m can be achieved in a 45 m visual space.

The paper is organized as follows: Previous studies are reviewed in Section 2. The qualitative distance and relative direction functions are given in Section 3. The method of positioning localities is provided in Section 4. Examples are presented in Section 5, followed by a discussion in Section 6. Conclusions are provided in Section 7.

## 2. Related Work

Related works on positioning locality with locality description in existing literature are briefly presented in this section.

### 2.1. Locality Description

Locality description answers a “where” question. As a predominant method of human spatial communication, locality description reflects human spatial cognition and contains a considerable amount of vague positional information [8,17]. Guo [18] argued that locality description contains reference objects (ROs), which refer to any named features in locality description, and their related spatial relations. The spatial relations in locality description play an important role in positioning locality. Wieczorek [19] described a method to combine all the types of uncertainty into a point radio to georeference locality description. When considering the shape of the ROs, Liu [10] proposed a general probabilistic method for positioning locality, which can combine a set of uncertain spatial relations (e.g., distances and directions). Zhou [20] described a conceptual model of fingerprints from locality descriptions by landmarks to capture the concept of place in human perception.

The locality description contains at least one RO and its related spatial relation. It may also be complex, linking different references by spatial relationships, either explicitly as “I am near object A, and it is in front of me”, or implicitly as “Object A is in front of me”, implying “Object A is near to me, and it is in front of me” [11,17].

### 2.2. Landmarks

Landmarks play a crucial role in human spatial cognition, whether as a navigational aid or a locality description [20,21]. As the first step of spatial knowledge acquisition, landmarks, which have attracted the interest of many researchers, play a crucial role in the acquisition and the representation of human spatial knowledge in daily life [21,22,23]. In spatial cognition, landmarks represent a cluster of objects at a high level and serve as ROs (anchor) to locate the target object (TO) [22].

The characteristics of landmarks include prominence and prototypicality [23]. Conventional work on landmark extraction is mainly based on questionnaires, which are cumbersome and labor intensive [24]. On the basis of the characteristics of landmarks, many scholars have used the saliency model to extract landmarks in different scenes [21,25]. Tezuka [25] extracted small-scale landmarks from digital documents by using a web mining approach. 

The selection of landmarks also depends on context [24]. As a navigational aid, landmarks provide orientation cues and verify route progress. Caduff [21] argued that three factors, namely, degree of differentiation, visual access, and complexity of spatial layout, contribute to the saliency of landmarks. They can be regarded as points in a small scale or as polygons in a large scale. Lyu [26] proposed indicators to develop a computational indoor landmark extraction method. Zhu [27] provided a method to compute the saliency of the POIs (points of interest), i.e., shops, to extract indoor landmarks. When compared with navigation, all kinds of landmarks can play a greater role in the context of locality description [20]. Therefore, the concept of landmarks is based on all kinds of indoor POIs (i.e., shops). The related definition is provided in Section 3.1.

### 2.3. Spatial Relations: Distance and Direction Relationship

Spatial relations can be divided into topological, distance, and direction relations. When compared with distance and direction relations, topological relation conveys more fuzzy positioning information, and other relations can reflect it to some extent. Distance and direction relations in locality description are generally used together for positioning locality.

Distance relationship can be categorized as qualitative and quantitative. Quantitative distance is the numeric distance value in practice, which is also called semi-qualitative, because of its uncertainty. Different uncertainties cause different probability distributions, such as formal and normal distribution [10]. Qualitative distance (e.g., near) is used more frequently than quantitative distance in locality description [11]. As one of the most fundamental spatial cognitive distance, the vague spatial relation “near” attracts many scholars’ attention [28,29]. Worboys [28] conducted a cognitive experiment in a university campus to explore how humans think about the vague spatial relation of nearness in the context of environmental space and found that the relation between conceptual distance and Euclidean distance conforms to a general S-curve. When considering context factors, Yao [29] presented ordered logit regression to predict the relationship between linguistic (e.g., near) and metric distance measures. In contrast to the cognitive aspects above, Martin [13] argued that the near relation can be modeled with a Voronoi diagram in computational geometry. Gong [12,14] defined a mixed probability function, which is based on Euclidean distance and Voronoi stolen area to address the near relation for points. Inspired by this idea, we will extend this function to polygon landmarks. Details are presented in Section 3.1.

**Figure 1:**
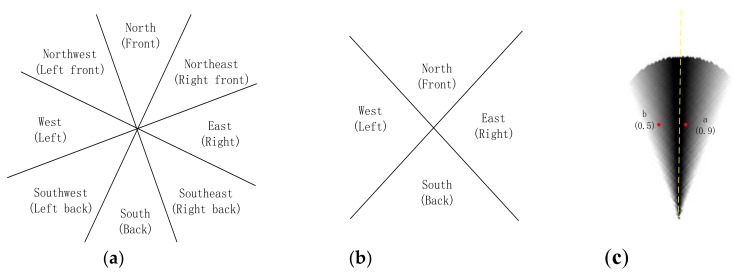
(**a**) Eight-cone based model; (**b**) four-cone based model; and, (**c**) probability distribution in the cone-based model (the probability of point a (0.9) is greater than that of b (0.5) in the direction).

**Figure 2:**
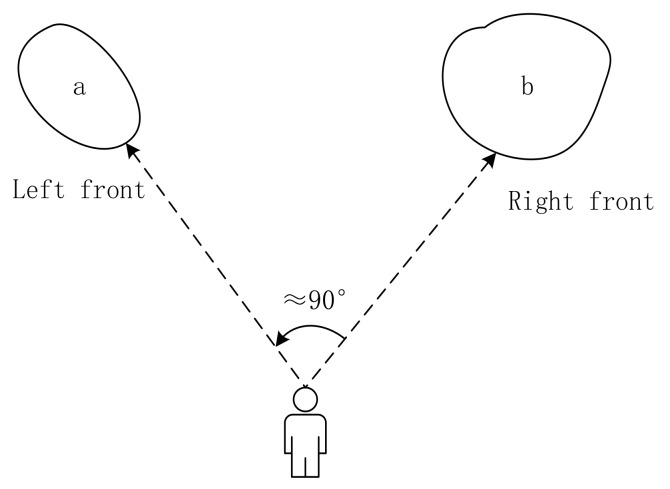
Illustration of relative direction (the angle that turns from right front to left front is nearly 90°).

Direction relationship can be divided into absolute and relative directions. Absolute direction relationships are used more frequently outdoors, where humans have a good sense of direction. The spatial space can be divided into four or eight cones, according to different contexts [30] (Figure 1a,b). The greater distance between an object and the center line of its cone corresponds to a lower probability that the object owning to its direction [10] (Figure 1c). In contrast to absolute direction, relative direction relations are frequently used in situations where humans may have a poor sense of direction, such as indoors. Krishnapuram [15] argued that human perception between two objects is closely related to angular information (Figure 2), and the distance between the people and the object is unimportant. On the basis of this idea, he defined the relative direction membership function, that is, left of, right of, above, and between. Extending the “between” relation into a medical image, Bloch [31] defined a fuzzy notion of visibility. Many relative relations have been developed since then [32,33,34].

## 3. Membership Functions Based on Fuzzy Set: Near and Relative Direction

The fuzzy spatial relations, that is, near and relative direction relations used in locality description indoors, will be introduced in this section. Their membership functions are conducted based on fuzzy set.

### 3.1. Membership Function for Near Relation

**Definition** **1.** ***Landmark:*** *POI (i.e., shop) which is polygon indoors.*

Any features in space can be called POI. To focus on our method, the shop data are available and should be regarded as polygon indoor.

For accuracy, all of the ROs in locality description are landmarks, and the positioning locality is called TO.

**Figure 3:**
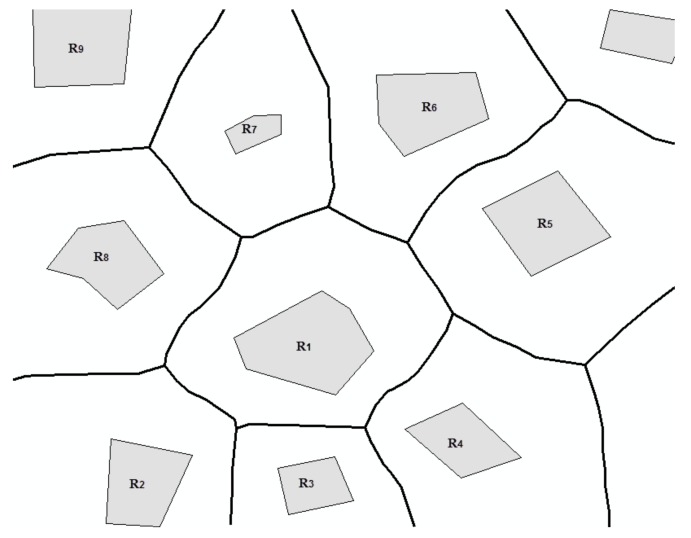
Neighbors of R_1_.

**Definition** **2.** ***Neighbors of RO R:*** *A set of spatial entities (points or polygons) share a common edge of Voronoi diagram neighbor with each other. We denote neighbors of R with neigh(R). As shown in Figure 3, neigh(R_1_) = {R_2_, R_3_, R_4_, R_5_, R_6_, R_7_, R_8_}.*

The position of R can be described by its neighbors. If a site (TO) is inserted into the space, then it can be described and positioned by one or several of its neighbors.

**Definition** **3.** ***Neighboring area of RO R:*** *The area of R that a site (TO) can be inserted into and be described by or neighbors R. The neighboring area of R is denoted as NeighArea(R).*

**Figure 4:**
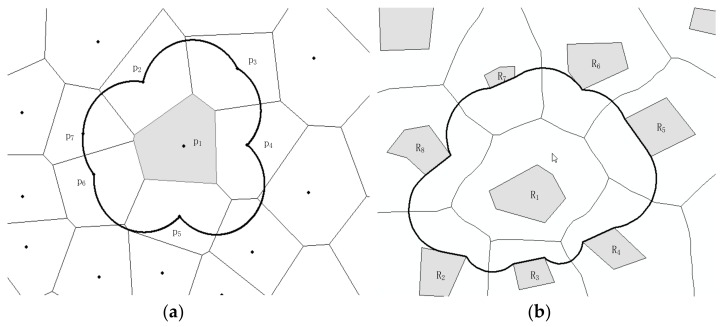
Neighboring area of (**a**) point p_1_ and (**b**) polygon R_1_.

The center of the circumcircle of Delaunay triangulation is the vertex of its related Voronoi. The dual graph of Voronoi diagram corresponds to the Delaunay triangulation. For point set {p_1_, p_2_, p_3_, p_4_, p_5_, p_6_, p_7_}, of which neighbors(p_1_) = {p_2_, p_3_, p_4_, p_5_, p_6_, p_7_}, the Delaunay triangulations for p_1_ and its neighbors are formed. The neighboring area of p_1_ is the union of the circumcircle of its Delaunay triangulation, whose boundary consists of circle arcs (Figure 4a). The neighboring area extends to polygon RO R_1_, of which neighbors (R_1_) = { R_2_, R_3_, R_4_, R_5_} and NeighArea(R_1_) are shown as Figure 4b.

**Figure 5:**
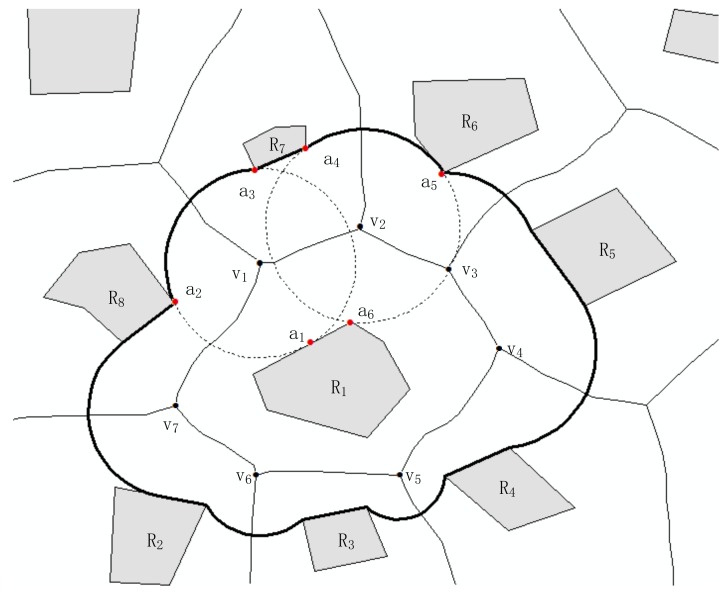
Illustration of the process of obtaining Nerg*Area*(R_1_).

The process of obtaining neighboring area of RO R_1_ is as follows (Figure 5): For ROs in space, R_1_ is neighbor to R_2_, R_3_, R_4_, R_5_, R_6_, R_7_, and R_8_. The vertices of the Voronoi polygon of R_1_ are v_1_, v_2_, v_3_, v_4_, v_5_, v_6_, and v_7_. The vertex v_1_ is the common vertex of R_8_, R_7_, and R_1_. The nearest points of R_7_, R_8_, and R_1_ to v_1_ are a_1_, a_2_, and a_3_, respectively. The circumcircle of triangulation with vertices a_1_, a_2_, and a_3_ is drawn, and the arcs a_2_a_3_ between R_8_ and R_7_ are obtained. Other arcs between ROs are obtained, and the arcs are connected with segments of ROs (e.g., segment of R_7_ a_3_a_4_) to form a closed cycle.

**Figure 6:**
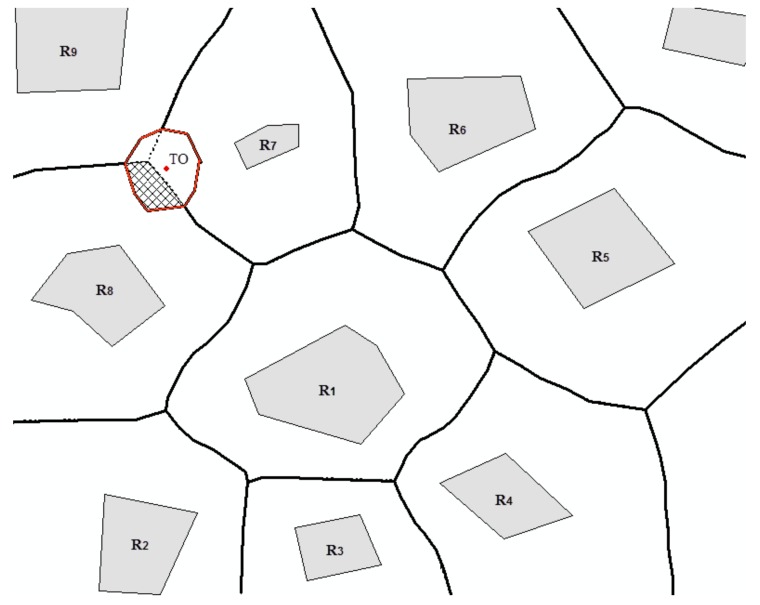
Illustration of stolen area (the area delineated by a red line is the area stolen from R_7_, R_8_, and R_9_; the dashed area is the area stolen from R_8_).

**Definition** **4.** ***Stolen area:*** *When a site (TO) is inserted into the existing Voronoi diagram of ROs, the stolen area is the area that is part of the Voronoi region of the original RO but now belongs to the Voronoi region of TO (Figure 6).*

On the basis of Euclidean distance and the stolen area [12,14], the membership function for near relation is defined as P_near_(i, R_i_):

(1) pnear(t,Ri)=Aimind(t,Ri)2∑Rk⊂neigh(t)Akmind(t,Rk)2

The equation is based on fuzzy set, which maps the near relation to the interval [0, 1] and it reflects the degree of near relation. In Equation (1), t represents TO, t∈NeighArea(R_i_), and min d(t, R) is the squared minimum distance between t and R_i_. A_k_ represents the area stolen from R_k_ by t.

### 3.2. Relative Direction Membership Function

When an individual turns around between two ROs, such as from front to right, the cone that is searched relates to angular information. On the basis of this concept, we define the eight-cone (front, left, right, back, front–left, front–right, back–left, and back–right) relative direction membership function p_reldir_(Θ):

(2) preldir(Θ)={1π8+|π4×path(Θ)−Θ|π8−a0  |π4×path(Θ)−Θ|≤aa≤|π4×path(Θ)−Θ|≤π8|π4×path(Θ)−Θ|≥π8

If the space is divided into 4 cones, i.e., front, left, right, back, the Equation (2) can be revised as Equation (3).

(3) preldir(Θ)={1π4+|π2×path(Θ)−Θ|π4−a0  |π2×path(Θ)−Θ|≤aa≤|π2×path(Θ)−Θ|≤π4|π2×path(Θ)−Θ|≥π4

**Figure 7:**
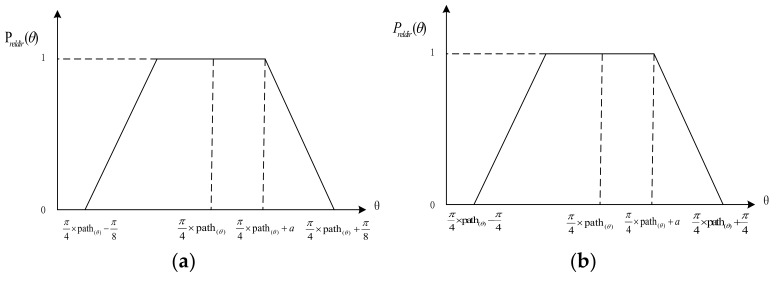
Illustration of relative direction membership function. (**a**) Equation (2); and, (**b**) Equation (3).

The illustration of relative direction membership functions are provided in Figure 7. The parameter Θ is the angle turning from one direction to other direction. To get an optimistic result, the value for *a* can be adjusted according to reality.

**Figure 8:**
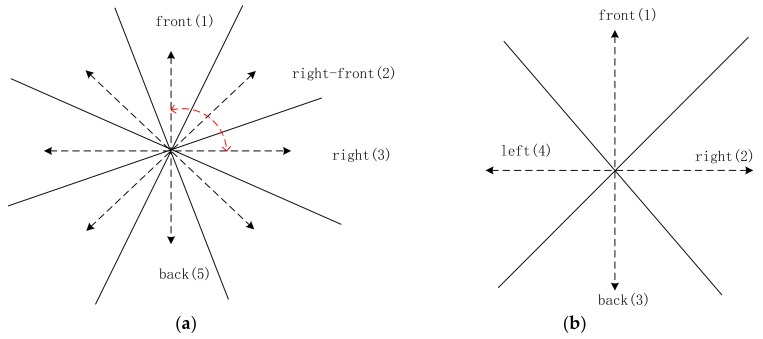
Illustration of path_(Θ)_. (**a**) eight sectors and the path_(Θ)_ is marked with a red dashed line; and, (**b**) four sectors.

The parameter path_(Θ)_ in Equations (2) and (3) is the minimum path between the center lines of corresponding cones. As shown in Figure 8a,b, the visual field is divided into eight sectors (front, back, left, right, right–front, right–back, left–front, and left–back) for Equation (2) and four sectors (front, right, back, and left) for Equation (3). The dashed lines are the center lines of the corresponding cones. Each center line is assigned a number clockwise (e.g., front is assigned 1). In Figure 8a, from front (1) to right (3), path_(Θ)_ = 2.

## 4. Method

In this section, the locality positioning method is introduced and described in detail, as follows (Algorithm 1):


**Algorithm 1:** Algorithm for positioning with direction and near relations
Obtain the domain where the positioning localities may be located. (Section 4.1) Calculate the probability of relative direction in the domain, i.e., P_reldir_. (Section 4.2) Calculate the probability of qualitative distance (“near”) in the domain, i.e., P_qdis_. (Section 4.3) Calculate the locality using a joint probability function which consist of qualitative distance and relative direction function. (Section 4.3) **End for**


Locality description generally contains three ROs at most, but positioning localities when the locality description contains only one RO is impossible. Hence, we divide the situation of positioning locality into two scenes: ***Scene 1***, locality description with two ROs, and ***Scene 2***, locality description with three ROs. Some differences are noted in the method for the two scenes, and the details are introduced in the following sections.

### 4.1. Domain of Positioning Localities

**Definition** **5.** ***Domain of TO t:*** *Domain where TO t may locate. We denote it with Domain(t).*

A site (*TO t*) can be described by its neighbors. As stated in Section 3.1, the neighboring area of R is that a site (*TO* *t*) can be a neighbor and can be described by R. If a *TO* *t* is described with R_i_ (i = 1, 2, 3), then Domain(t) = ∪NeighArea(R_i_). No difference is noted for *Scenes 1* and *2*.

### 4.2. Probability of Relative Direction in Domain

This section first provides a definition for the calculation of relative direction. Next, the method of calculating the probability of relative direction in domain is proposed for two different scenes.

**Figure 9:**
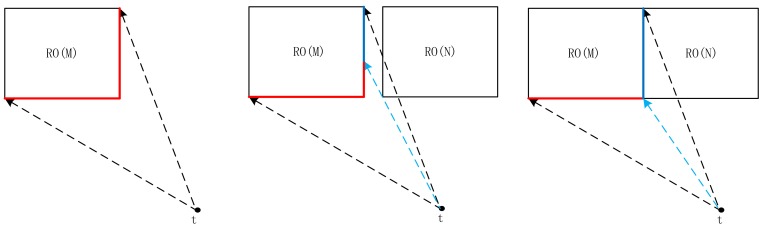
Definition of visible segment Visible_Seg(M) (red line). The red and blue solid lines form the boundary of reference object (RO) M from a locality *t*. The blue solid line is the invisible segment, and the red solid line is the visible segment. The lines of sight are simulated by dashed lines, of which the blue dashed line is the auxiliary line (**a**) whole part; (**b**) interrupted by adjacent RO N; and, (**c**) interrupted by disjoint RO N.

**Definition** **6.** **Visible Segment:** The segment boundary of a landmark is observed from a locality that is consistent with spatial cognition (Figure 9).

**Figure 10:**
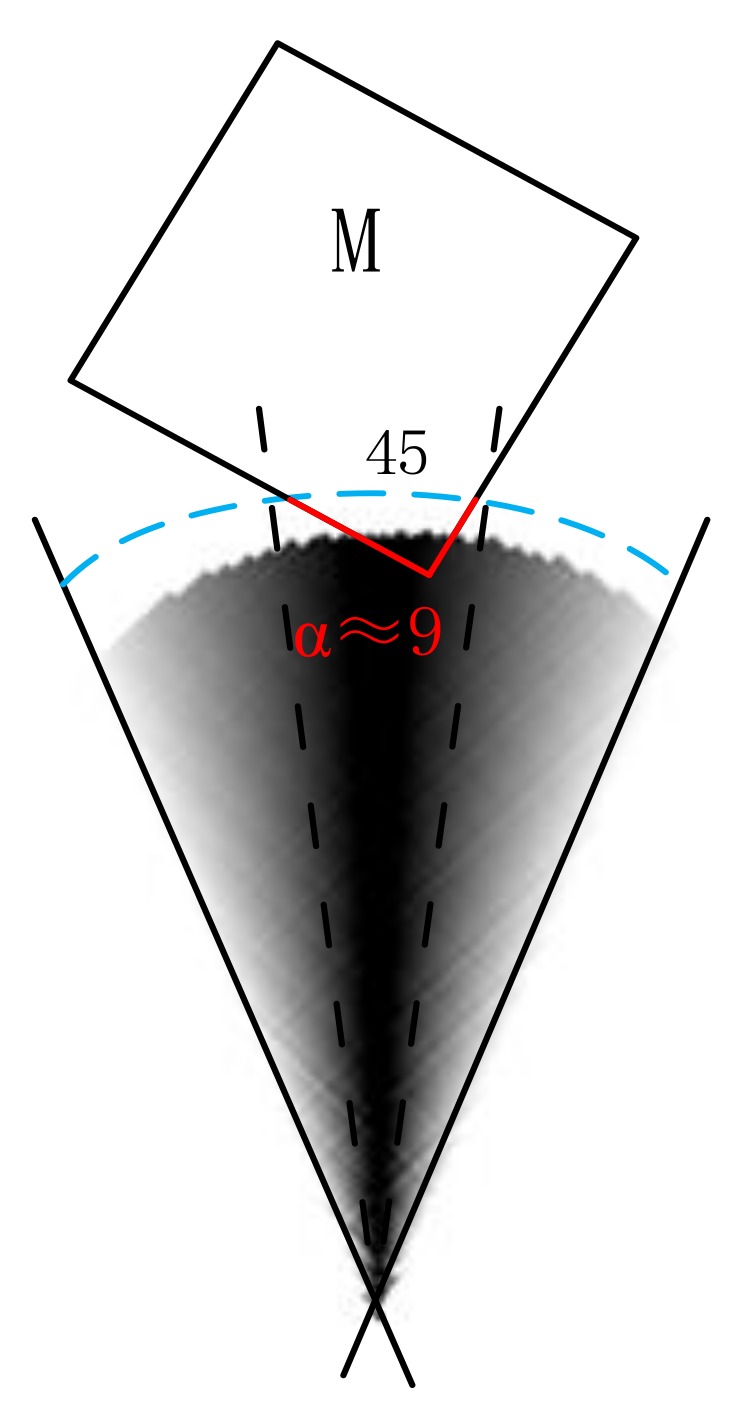
Illustration of Pareto principle for visible segment; the red line of the visible segment meets the Pareto principle.

The visible segment should meet not only the characteristic of visibility, but also the Pareto principle that states that roughly 80% of effects originate from 20% of the cause, whether from an algorithmic or spatial cognitive perspective. As shown in Figure 10, if the space is conducted into eight cones, namely, front, back, left, right, right–front, right–back, left–front, and left–back, then the angle of each cone is 45°. The occupation angle of the portion of the visible segment in the cone should be approximately 9°.

We assume polygon (RO or Domain(t)) has a set of points, namely, A = {a_1_, a_2_, …, a_n_}. For a ∈ Visible_Seg(A), b ∈ Visible_Seg(B), and t ∈ Domain(t), we let dir(A, t, B) denote the angle between point t to RO A and point t to RO B. The process of calculating relative direction probability, P_reldir(t)_, for two scenes is as follows:

***Scene 1: Two ROs A and B***

(4) $$P_{\mathrm{reldir}}{}_{\left(t\right)}=\frac{P_{\mathrm{dir}(A,t,B)}}{\sum_{i\in \mathrm{Domain}(t)}P_{\mathrm{dir}(A,i,B)}}$$

P_dir_(A, t, B) is the membership degree that maps the dir(A, t, B) by using the relative direction membership function Equation (2).

***Scene 2: Three ROs A, B, and C***

(5) $$P_{\mathrm{reldir}}{}_{\left(t\right)}=\frac{P_{\mathrm{dir}(A,t,B)}P_{\mathrm{dir}(A,t,C)}P_{\mathrm{dir}(B,t,C)}}{\sum_{i\in \mathrm{Domain}(t)}P_{\mathrm{dir}(A,i,B)}P_{\mathrm{dir}(A,i,C)}P_{\mathrm{dir}(B,i,C)}}$$

P_dir_(A, t, B), P_dir_(A, t, C), and P_dir_(B, t, C) are the membership degrees that map dir(A, t, B), dir(A, t, C), and dir(B, t, C) via the relative direction membership function Equation (2).

### 4.3. Probability of Qualitative Distance in Domain

If TO t, t ∈ Domain(t) is described with R, then the qualitative distance probability of t, and P_near_(t, R) can be computed according to Equation (1). The process of calculating qualitative distance probability, P_qdis(t)_, for two scenes is as follows:

***Scene 1: Two ROs A and B***

(6) $$P_{\mathrm{qdis}}{}_{\left(t\right)}=\frac{P_{\mathrm{near}(t,A)}P_{\mathrm{near}(t,B)}}{\sum_{i\in \mathrm{Domain}(t)}P_{\mathrm{near}(i,A)}P_{\mathrm{near}(i,B)}}$$

***Scene 2: Three ROs A, B, and C***

(7) $$P_{\mathrm{qdis}}{}_{\left(t\right)}=\frac{P_{\mathrm{near}(t,A)}P_{\mathrm{near}(t,B)}P_{\mathrm{near}(t,C)}}{\sum_{i\in \mathrm{Domain}(t)}P_{\mathrm{near}(i,A)}P_{\mathrm{near}(i,B)}P_{\mathrm{near}(i,C)}}$$

### 4.4. Positioning Localities

The positioning localities can be calculated by a joint probability, which consists of qualitative distance probability and relative direction probability. Let P_(t)_ represent the probability of positioning localities, t ∈ Domain(t). The positioning locality is the maximum probability point or the center point of the maximum probability in the domain. The equations for the two scenes are the same, that is, Equation (8).

(8) $$P_{\left(t\right)}=\frac{P_{\mathrm{reldir}(t)}P_{\mathrm{qdis}(t)}}{\sum_{i\in \mathrm{Domain}(t)}P_{\mathrm{reldir}(i)}P_{\mathrm{qdis}(i)}}$$

However, two positioning localities appear for Scene 1, where an angle between two directions that is not equal to 180° is unacceptable. A principle for obtaining a unique positioning locality is defined.

**Figure 11:**
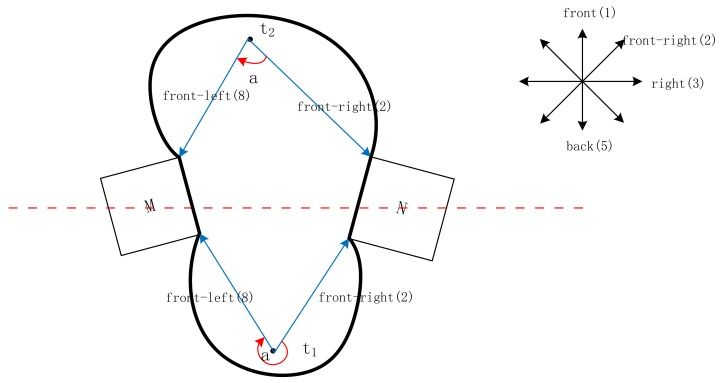
Illustration of the principle (the thick black lines correspond to the Domain(t); solid blue lines represent the direction lines; and, the direction of rotation is marked with a solid red line).

**Principle:**  We assume that the scene of locality description is as follows: “*My front–right is N, and my front–left is M*”. As shown in Figure 11, eight directions from front to front–left clockwise are assigned corresponding numbers from 1 to 8. The path(a) is the path between two direction lines. The two positioning localities on the two sides of the line connecting the ROs are t_1_ and t_2_. Lines 8 and 2 connect M and N to the positioning locality (i.e., t_1_ and t_2_), respectively. The unique positioning locality should meet the requirement that the direction from front–right (2) to front–left (8) is clockwise, and path_(a)_ *= 6*, that is, t_1_.

## 5. Case Study

A cognitive experiment is conducted in a shopping market indoors with a sufficient number of participants. The participants include males and females with different backgrounds. Their ages range from 20 to 55, and they have normal spatial cognition. 

Locality description is complex, and human tends to use different spatial relations to description locality in different context. To simulate the realty and focus on the method, a description of context is given and is told to the participants. Meanwhile, before locality description, the participants are told to look around.

**Description of the context:**  When you lose track of your friends or family, your family calls you “Where are you?” Imaging that there is a phone can translate your locality description into localities. Then, your friends or family can find you easily. You can describe your locality with distance (e.g., near) or directions.

**Example** **1.** A representative test ground that meets the positioning method should be selected. Under the described context, the random participants in the shopping market are told to look around and to describe their localities with distance or directions. We record the participants’ locality description and localities.

**Figure 12:**
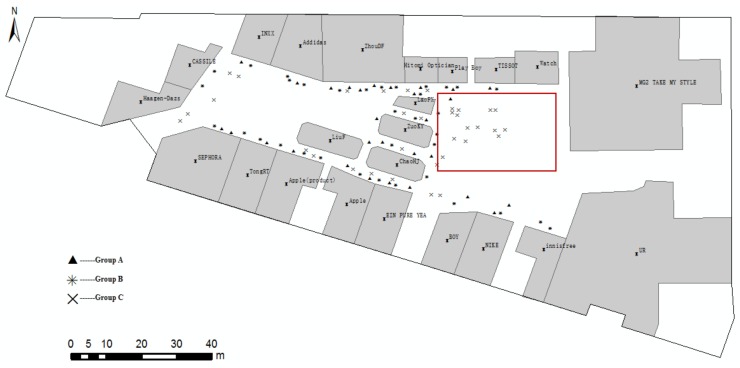
Distribution of localities of participants.

As shown in Figure 12, the localities of participants are marked as points. We analyse the distribution of locality description and divide them into three groups (Figure 12): Group A, locality description only with near; Group B, locality description with one direction; Group C, locality description with more than one direction. Group C, meeting the positioning method, tends to locate in a place that is spacious and has more landmarks. From the above analysis, the locations of groups A, B, and C tend to consist of human spatial cognition and expression.

On the basis of Example 1 and the focus on our method, we conduct our cognitive experiment in a spacious area (i.e., red line enclosed region in Figure 12) and divide the collected data into two scenes, according to the RO number. For the calculation, the range of parameter *a* in Equation (2) is [2,5] multiplied by path_(Θ)_. Without additional contextual information, we cannot tell which cone-based model the direction relationship “left” stands for. But, “front-right” stands for eight cone-based model. So that, we use four cone-based model when lacking contextual information. The angle value that meets the Pareto principle in the visible segment should be roughly 10° and 20° for the eight and four cone-based models, respectively. All of the parameters can be adjusted according to different realities.

**Figure 13:**
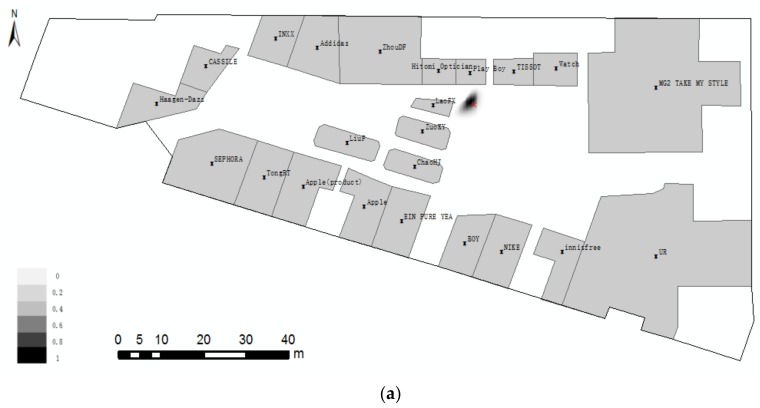

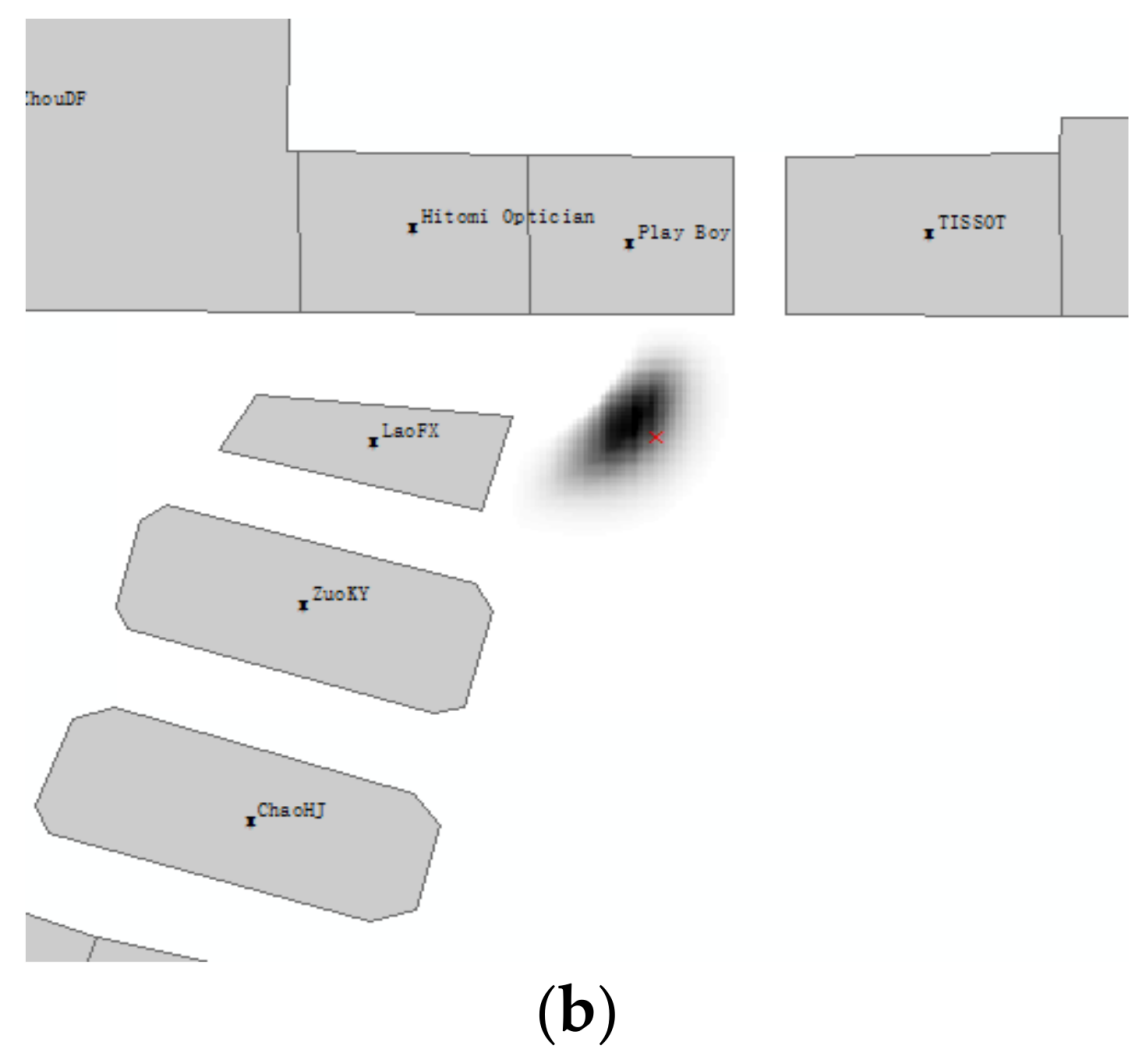
Positioning with two ROs. The locality description is “Front is PlayBoy, left is LaoFX” (**a**) global and (**b**) local.

**Example** **2.** As shown in Figure 13, the locality description for Scene 1 is ”Front is PlayBoy, left is LaoFX”. Figure 13b shows a local map for the locality description. A darker color corresponds to an increased probability that it is the positioning locality.

**Figure 14:**
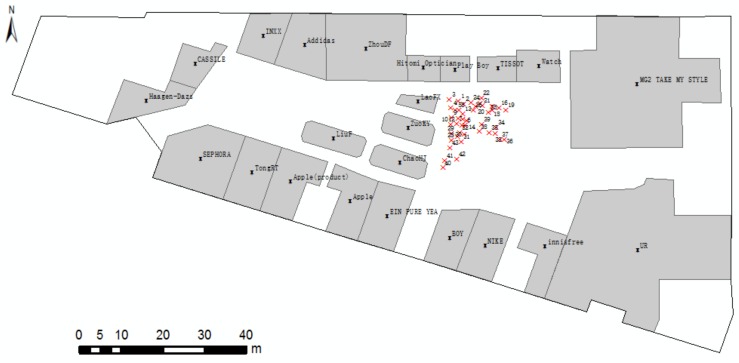
Positioning errors with two ROs.

**Figure 15:**
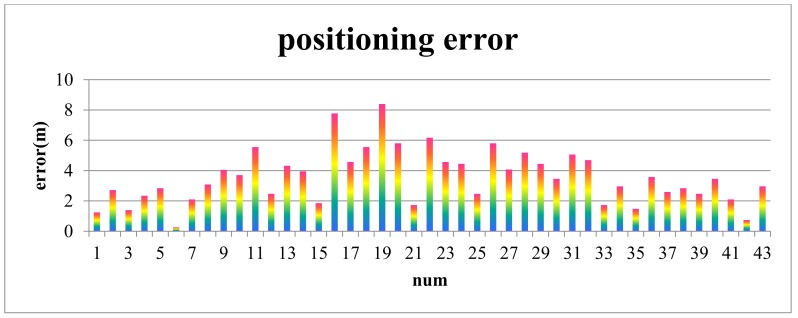
Positioning errors with two ROs.

A group cognitive experiment is conducted to estimate the positioning accuracy for Scene 1, and related data are presented in Table 1. The sample data distribute randomly and uniformly in the space (Figure 14). The numbers in Figure 14 correspond to the numbers in the Table 1. The positioning error, which is the distance from the maximum probability point or the center point of the maximum probability to the locality of the participant, is shown in Figure 15.

As shown in Figure 15, the maximum and minimum positioning errors are 8.39 and 0.26 m, respectively, and the mean positioning error is 3.55 m.

**Figure 16:**
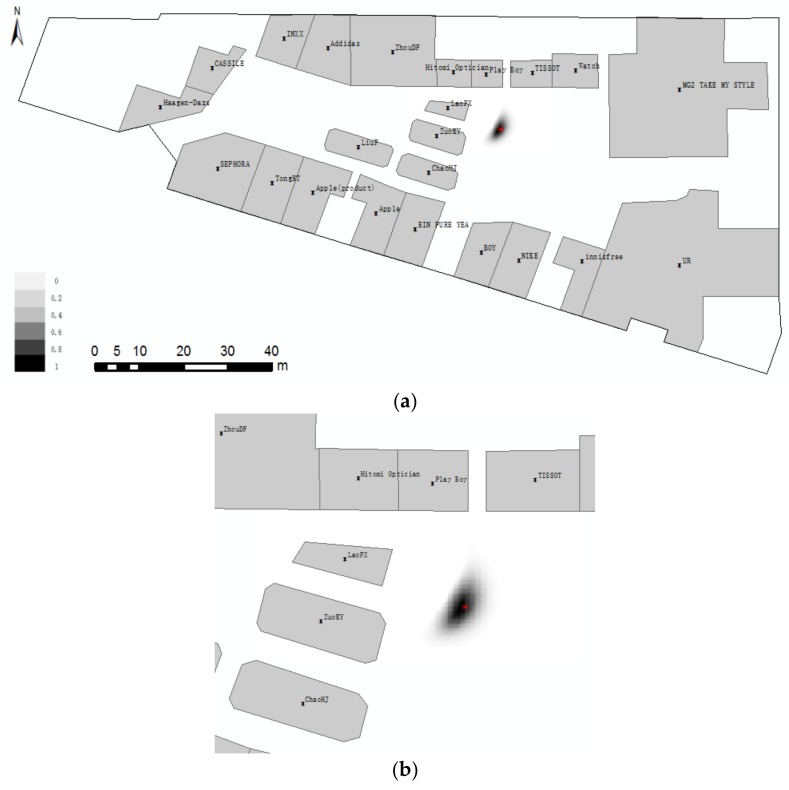
Positioning with three ROs. The locality description is “Front is LaoFX, front–left is ZuoKY, and front–right is PlayBoy” (**a**) global and (**b**) local.

**Example** **3.** As shown in Figure 16, the locality description for Scene 2 is ”Front is LaoFX, front–left is ZuoKY, and front–right is PlayBoy”. Figure 16b shows a local map for the locality description.

**Figure 17:**
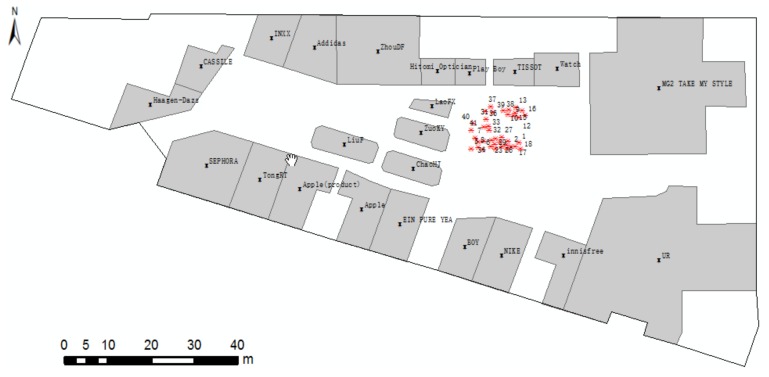
Positioning errors with three ROs.

**Figure 18:**
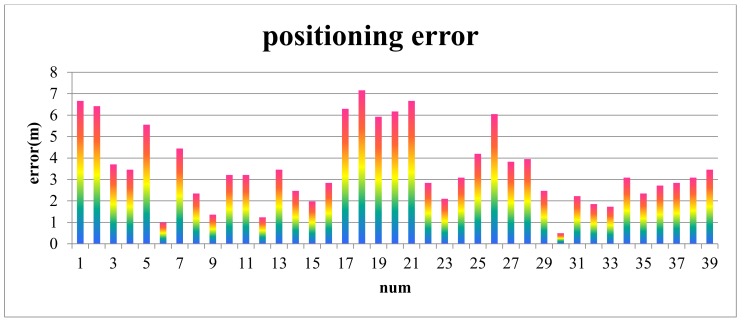
Positioning errors with three ROs.

A group cognitive experiment is conducted to estimate the positioning accuracy for Scene 1, and related data are presented in Table 2. The sample data distribute randomly and uniformly in the space (Figure 17). The numbers in Figure 17 correspond to the numbers in the Table 2. The positioning error, which is the distance from the maximum probability point or the center point of the maximum probability to the locality of the participant, is shown in Figure 18.

As shown in Figure 18, the maximum and minimum positioning errors are 7.16 and 0.49 m, respectively, and the mean positioning error is 3.54 m.

Locality description reflects human spatial cognition, which has both commonness and personality. More people in the adjacent place describe locality with the same ROs and even the same directions (Table 1, numbers 27–31). The existing individuals in the adjacent place describe locality with different ROs (Table 1, numbers 17 and 18), and even different directions (Table 1, numbers 11 and 14). This difference can be explained by the habits or standing orientations when describing locality. Another characteristic of spatial cognition is uncertainty, which reflects personality to the same extent. Under the naïve cognition of the complex environment, the positioning accuracy does not exceed 3.55 m and is more acceptable than a 3–5 m positioning accuracy using common smartphones with complex and costly indoor positioning techniques.

Context is an important factor in positioning locality with locality description. If more contexts (e.g., spatial and semantic) are available, then positioning accuracy improves. (1) Semantic context: aside from the locality description with two or three ROs with directions, other ROs that can provide additional position clues, such as “near marble columns” may appear in locality description. All of these clues can refer to our model or other related models. (2) Spatial context: the infrastructures in the domain may affect the probability distribution.

## 6. Discussion

In this section, we will have a deeper discussion with respect to (1) positioning errors and (2) near relation.

### 6.1. Positioning Errors

For fuzzy spatial cognition, positioning errors with locality description are inevitable. Positioning errors can be divided into two aspects according to position clues, namely, direction and near relation. (1) Locality description of adjacent localities occurs at different angles. In Table 1, numbers 11 and 14, the angles of locality description are 90° and 45°, respectively. This result could be explained by the standing orientations or the attractive part of ROs. We calculate the direction by using the visible segment of ROs from the general; (2) Near, a relative conception, reflects distance only. In ILRS, more people select near ROs, but some people select relatively far ROs to describe locality, resulting in many positioning errors. As shown in Table 1, numbers 16 and 19, LaoFX, which is not near when compared with other ROs, such as PlayBoy or Watch, is selected. The above two aspects are expressions of spatial fuzzy and naïve cognition.

### 6.2. Analysis of Near Relation

As a supplement of quantitative distance, qualitative distance is used frequently in locality description. When compared with positioning locality indoors with quantitative distance, the positioning accuracy with qualitative distance (3.5 m) [35] is more acceptable, in contrast to intuitive cognition where the distance with number has greater accuracy.

The qualitative distance relation model stated in the paper, that is, near, is proposed based on then Voronoi diagram for polygons, which is an important tool for modeling spatial problems. The generation of a Voronoi diagram for polygons can be raster- or vector-based [36,37]. The vector-based algorithm is not efficient and cannot be integrated in the GIS software [37]. Our proposed algorithm is raster-based, which is simple, but it has a slow process. Increased attention should be given to the development of an efficient algorithm for generating a Voronoi diagram for polygons or other features.

In addition, the weights of all the landmarks indoors are the same, as follows: (1) The factors (i.e., size, height) that affect the distance cognition of landmarks indoors are almost the same; (2) Locality description has no substitute for the weight of landmarks. If the weights of all the landmarks are different, then the near distance relation based on ordinary Voronoi diagram is infeasible. Whether the multiplicatively weighted Voronoi diagrams can answer this question is unknown, and this issue will be included in our future work.

## 7. Conclusions

Implicitly is an inherent characteristic of locality description, especially in ILRS. Based on this, we propose that near landmarks are selected easily when describing locality with the directions of locality description. To achieve positioning of localities with directions description in ILRS, we propose a joint probability function that consists of qualitative distance (i.e., near relation) and relative direction membership function. The qualitative distance membership function that considers both minimum Euclidean distance and the stolen area is based on fuzzy set. For consistency with cognition, some definitions are provided during the calculation of relative direction, which can also reduce the number of points to be explored from an algorithmic point of view. Some cognitive experiments are conducted and demonstrate that a positioning accuracy of 3.55 m can be achieved within a 45 m visual space in ILRS.

The membership function for near relation proposed in our paper is raster-based, which has low efficiency. In our future work, vector-based or parallel algorithm will be developed, which is helpful for enhance the algorithm efficiency. Furthermore, the function is based on simple geometric calculation and does not consider contextual information, such as personal reputation, background, and hobbies, which are important to distance cognition. If enough data are available, then the near relation can be modeled based on ordered logit regression (OLR) or SVM.

Our method is based on spatial cognition, so that reasonable direction cognition is necessary. Despite the fact that the relative direction calculation based on visible segment in our work performances well in positioning locality, it does not work well in all cases. For instance, if the length of visible segment of one RO is much longer that the other RO, which is rare indoor, whether our method is feasible should be further discussed.

## Figures and Tables

**Table 1:** Locality description with two ROs.

Num	RO1		RO2	
	Name	Direction	Name	Direction
1	PlayBoy	front	LaoFX	left
2	LaoFX	front-left	PlayBoy	front-right
3	PlayBoy	front	LaoFX	left
4	LaoFX	front	PlayBoy	right
5	LaoFX	front	PlayBoy	right
6	ZuoKY	front-left	LaoFX	front-right
7	ZuoKY	front-left	LaoFX	front-right
8	PlayBoy	front	LaoFX	left
9	LaoFX	front	ZuoKY	left
10	ZuoKY	front-left	LaoFX	front-right
11	LaoFX	front-left	PlayBoy	front-right
12	LaoFX	front-right	ZuoKY	front-left
13	PlayBoy	front-right	LaoFX	front-left
14	LaoFX	front-left	PlayBoy	front
15	ZuoKY	front-left	LaoFX	front-right
16	LaoFX	left	TISSOT	front
17	PlayBoy	front-left	TISSOT	front
18	LaoFX	left	TISSOT	front
19	LaoFX	left	TISSOT	front
20	PlayBoy	front-right	LaoFX	front-left
21	PlayBoy	front	TISSOT	left
22	TISSOT	front	PlayBoy	front-left
23	PlayBoy	front-left	TISSOT	front
24	LaoFX	front-left	PlayBoy	front-right
25	PlayBoy	front-left	TISSOT	front
26	LaoFX	front-left	PlayBoy	front-right
27	ZuoKY	front	LaoFX	front-right
28	LaoFX	front-right	ZuoKY	front
29	ZuoKY	front	LaoFX	front-right
30	ZuoKY	front	LaoFX	front-right
31	LaoFX	front-right	ZuoKY	front
32	ZuoKY	left	LaoFX	front
33	TISSOT	left	ZuoKY	front
34	ZuoKY	front	TISSOT	left
35	ZuoKY	front	TISSOT	left
36	ZuoKY	left	TISSOT	front
37	TISSOT	front	ZuoKY	left
38	TISSOT	front-right	LaoFX	front-left
39	LaoFX	left	TISSOT	front
40	CHJ	front	ZuoKY	front-left
41	ZuoKY	front-left	CHJ	front
42	CHJ	front-right	ZuoKY	front-left
43	ZuoKY	front-left	CHJ	front-right

**Table 2:** Locality description with three ROs.

Num	RO1		RO2		RO3	
	Name	Direction	Name	Direction	Name	Direction
1	LaoFX	front-left	TISSOT	front	ZuoKY	left
2	ZuoKY	front	LaoFX	front-left	TISSOT	left
3	PlayBoy	front	LaoFX	front-left	ZuoKY	left
4	LaoFX	front	PlayBoy	front-right	ZuoKY	front-left
5	PlayBoy	front	LaoFX	front-left	ZuoKY	left
6	ZuoKY	front	LaoFX	front-right	CHJ	front-left
7	CHJ	front-left	LaoFX	front-right	ZuoKY	front
8	LaoFX	front-right	ZuoKY	front	CHJ	front-left
9	PlayBoy	front-left	TISSOT	front	Watch	front-right
10	TISSOT	front	PlayBoy	front-left	Watch	front-right
11	PlayBoy	front-left	Watch	front-right	TISSOT	front
12	TISSOT	front	PlayBoy	front-left	Watch	front-right
13	Watch	front-right	TISSOT	front	PlayBoy	front-left
14	PlayBoy	front-left	TISSOT	front	Watch	front-right
15	Watch	front-right	TISSOT	front	PlayBoy	front-left
16	Watch	front-right	TISSOT	front	PlayBoy	front-left
17	TISSOT	front	ZuoKY	left	LaoFX	front-left
18	LaoFX	front-left	TISSOT	front	ZuoKY	left
19	LaoFX	front-left	ZuoKY	left	TISSOT	front
20	TISSOT	front	LaoFX	front-left	ZuoKY	left
21	LaoFX	front-left	TISSOT	front	ZuoKY	left
22	ZuoKY	front	LaoFX	front-right	CHJ	front-left
23	LaoFX	front-right	ZuoKY	front	CHJ	front-left
24	ZuoKY	front	LaoFX	front-right	CHJ	front-left
25	ZuoKY	front	CHJ	front-left	LaoFX	front-right
26	LaoFX	front-left	TISSOT	front	ZuoKY	left
27	LaoFX	front-left	TISSOT	front	ZuoKY	left
28	ZuoKY	front	CHJ	front-left	LaoFX	front-right
29	ZuoKY	front	LaoFX	front-right	CHJ	front-left
30	LaoFX	front	PlayBoy	front-right	ZuoKY	front-left
31	PlayBoy	front	TISSOT	right	ZuoKY	left
32	LaoFX	front	ZuoKY	front-left	PlayBoy	front-right
33	PlayBoy	front-right	ZuoKY	front-left	LaoFX	front
34	LaoFX	left	TISSOT	front-right	PlayBoy	front-left
35	LaoFX	left	TISSOT	front-right	PlayBoy	front-left
36	Watch	front-right	TISSOT	front	PlayBoy	front-left
37	LaoFX	front-left	TISSOT	front-right	PlayBoy	front
38	LaoFX	front	PlayBoy	front-right	ZuoKY	front-left
39	PlayBoy	front-right	ZuoKY	front-left	LaoFX	front
